# Genome-wide transcriptional changes induced by phagocytosis or growth on bacteria in *Dictyostelium*

**DOI:** 10.1186/1471-2164-9-291

**Published:** 2008-06-17

**Authors:** Alessio Sillo, Gareth Bloomfield, Alessandra Balest, Alessandra Balbo, Barbara Pergolizzi, Barbara Peracino, Jason Skelton, Alasdair Ivens, Salvatore Bozzaro

**Affiliations:** 1 Department of Clinical and Biological Sciences, University of Turin, Ospedale S. Luigi, 10043 Orbassano, Torino, Italy; 2 Wellcome Trust Sanger Institute, Wellcome Trust Genome Campus, Hinxton, Cambridge, CB10 1SA, UK; 3 Division of Cell Biology, MRC-Laboratory of Molecular Biology, Hills Road, Cambridge, CB2 2QH, UK

## Abstract

**Background:**

Phagocytosis plays a major role in the defense of higher organisms against microbial infection and provides also the basis for antigen processing in the immune response. Cells of the model organism *Dictyostelium *are professional phagocytes that exploit phagocytosis of bacteria as the preferred way to ingest food, besides killing pathogens. We have investigated *Dictyostelium *differential gene expression during phagocytosis of non-pathogenic bacteria, using DNA microarrays, in order to identify molecular functions and novel genes involved in phagocytosis.

**Results:**

The gene expression profiles of cells incubated for a brief time with bacteria were compared with cells either incubated in axenic medium or growing on bacteria. Transcriptional changes during exponential growth in axenic medium or on bacteria were also compared. We recognized 443 and 59 genes that are differentially regulated by phagocytosis or by the different growth conditions (growth on bacteria vs. axenic medium), respectively, and 102 genes regulated by both processes. Roughly one third of the genes are up-regulated compared to macropinocytosis and axenic growth. Functional annotation of differentially regulated genes with different tools revealed that phagocytosis induces profound changes in carbohydrate, aminoacid and lipid metabolism, and in cytoskeletal components. Genes regulating translation and mitochondrial biogenesis are mostly up-regulated. Genes involved in sterol biosynthesis are selectively up-regulated, suggesting a shift in membrane lipid composition linked to phagocytosis. Very few changes were detected in genes required for vesicle fission/fusion, indicating that the intracellular traffic machinery is mostly in common between phagocytosis and macropinocytosis. A few putative receptors, including GPCR family 3 proteins, scaffolding and adhesion proteins, components of signal transduction and transcription factors have been identified, which could be part of a signalling complex regulating phagocytosis and adaptational downstream responses.

**Conclusion:**

The results highlight differences between phagocytosis and macropinocytosis, and provide the basis for targeted functional analysis of new candidate genes and for comparison studies with transcriptomes during infection with pathogenic bacteria.

## Background

Phagocytosis is the most ancient defense mechanism against microbes. It is already present in unicellular amoebae, where it also constitutes the major, if not unique, mechanism for uptake of nutrients [1]. In multicellular organisms with a developed immune system, phagocytosis is integrated in this system and is performed by professional phagocytes, such as macrophages, monocytes and PMN leukocytes, which are capable of ingesting and killing a large variety of microorganisms [2,3]. An additional function of phagocytosis in pluricellular organisms is non-inflammatory depletion of apoptotic cells.

Molecular mechanisms of phagocytosis are conserved throughout evolution. Important contributions to our understanding of phagocytosis have come indeed from utilization of genetically tractable organisms, such as *Dictyostelium discoideum *[1,4-9]. Several bacterial pathogens for humans, such as legionella, mycobacteria and salmonella, are pathogenic also for *Dictyostelium*, favouring the use of this model to study host-pathogen interactions [9-19].

In their natural habitat, *Dictyostelium *cells live as unicellular amoebae feeding on bacteria. Laboratory strains have been selected which grow on liquid nutrient media by fluid-phase endocytosis, though having retained the ability to phagocytose [20,21]. These strains are particularly useful for selecting mutants defective in phagocytosis, but able to grow by pinocytosis. They can also be used to assess changes in gene expression induced by phagocytosis, by shifting cells from axenic medium to bacterial culture, as done in the present work.

When challenged with bacteria or latex beads under shaking, axenically growing AX2 cells are able to phagocytose particles without any lag. This suggests that axenic cells possess the basic machinery for uptake of the bacteria. Several lines of evidence indicate, however, that adaptive changes occur upon cell incubation with bacteria: (1) the phagocytosis rate increases from 1 to 4 bacteria/min/cell during the first three hours of co-incubation [22,23]; (2) within 90 min from exposure to bacteria, the pattern of glycoproteins reactive to monoclonal antibodies against specific membrane sugars changes, resembling that of bacterially growing cells [24]; (3) some genes, such as the gene for the *vatB *subunit of the V-H+ ATPase or for the *eIF6 *factor, which is required for ribosomal assembly, are expressed at higher rates within 60 min of incubation with bacteria, when compared to axenic medium [25,26]; (4) cell doubling time diminishes from 8–10 hours, in axenic medium, to 4–6 hours during the first generation in bacterial culture and drops to 3 hours in the second generation (corresponding to the doubling time of bacterially-growing wild-type strains).

Thus, phagocytosis appears to rapidly elicit gene expression changes that possibly affect the efficiency of uptake, intracellular transport and digestion of the ingested particle and result in long lasting effects on metabolic pathways, cellular and membrane properties linked with phagocytosis and/or growth on bacteria.

In contrast to macrophages, *Dictyostelium *cells are easily amenable to molecular genetic analysis: the cells are stably transformed with both extrachromosomal or integrating vectors, gene disruption is favoured by the haploid genome, and several cell biological assays are available for selecting mutants [27]. The complete genome sequence has been made available recently [28], raising the possibility to use DNA microarrays to analyze transcriptional responses to phagocytic stimuli. Microarrays have been already used in *Dictyostelium *to study regulatory pathways during development [29-31] or host cell response during *Legionella *infection [32]. This is the first report on genome-wide transcriptional response to phagocytosis of and growth on non-pathogenic bacteria. For this purpose, DNA microarrays covering about 8.500 of the 12.500 genes present in the *Dictyostelium *genome have been used. This approach has led to the identification of 528 genes differentially expressed in cells exposed shortly to bacteria, 102 of which are shared by cells growing exponentially on bacteria. By excluding 85 genes, whose transcriptional changes may be due to both phagocytosis and growth on bacteria, 443 genes could be specifically regulated by phagocytosis. For some of the identified genes, a role in phagocytosis has been already demonstrated in the literature, for others such a role is plausible and can be further tested by generating deletion mutants. About two-third of the identified genes are down-regulated upon phagocytosis or growth on bacteria compared to axenic medium, suggesting that cell growth in artificial medium requires a larger number of genes to be more highly expressed.

## Results

### DNA microarrays, rationale and experimental design

In contrast to wild type cells, which are strictly dependent on phagocytosis for growth, selected axenic strains also grow on rich or minimal defined media, which are taken up by fluid-phase pinocytosis, mostly macropinocytosis [7]. At the level of single cells, bacterial uptake, phagosomal maturation and degradation occur within a few minutes, and can be followed as sequential steps by imaging techniques. At the cell population level, phagocytosis and metabolic adaptation to the new growth conditions are linked to each other in a continuum process, and cannot be cleanly separated from each other for the purposes of this paper, namely to study the transcriptional profile during phagocytosis. Changes in gene expression that may be induced by bacteria binding, uptake or intracellular vesicle traffic are intertwined with changes due to metabolic adaptation to growth on bacteria. Supplying bacteria directly to cells growing in axenic medium may reduce the effects due to different growth conditions, but will result in other variables, as the bacteria will rapidly grow in the rich medium and could release factors that affect *Dictyostelium *gene expression. Heat-killed bacteria are no valid alternative as they are taken up less efficiently than living bacteria and are degraded less rapidly, leading to engulfment and thus altering phagocytosis. Inert particles, such as latex beads have deleterious effects on the cells, because they are not digested and, though they are secreted, secretion is a very slow process, lasting a few hours after uptake. Engulfment and cell rounding up are the immediate effects.

We have tried to circumvent these problems, avoiding intentionally to interfere with the phagocytic process, and have chosen three experimental set-ups that allow indirectly to distinguish effects on gene expression due to phagocytosis from adaptation to growth on bacteria versus axenic growth. The experimental design, which is summarized in Table 1, is as follows.

**Table 1:** Experimental design and comparisons

Comparison	Cell strains and experimental conditions	Differentially expressed genes are:
**A**	axenic AX2 cells incubated on *E. coli *for 2 hours axenic AX2 cells incubated in medium for 2 hours	**upregulated**, if the log2 ratio of RNA expression values in nominator vs. denominator: ≥ 1
**B**	AX2 cells growing exponentially on *E. coli* AX2 cells growing exponentially in medium	
**C**	axenic AX2 cells incubated on *E. coli *for 2 hours AX2 cells growing exponentially on *E. coli*	**downregulated**, if the log2 ratio of RNA expression values in nominator vs. denominator: ≤ -1
**D**	V12M2 cells growing exponentially on *E. coli* AX2 cells growing exponentially on *E. coli*	

#### (1) Comparison A

**Figure 1:**
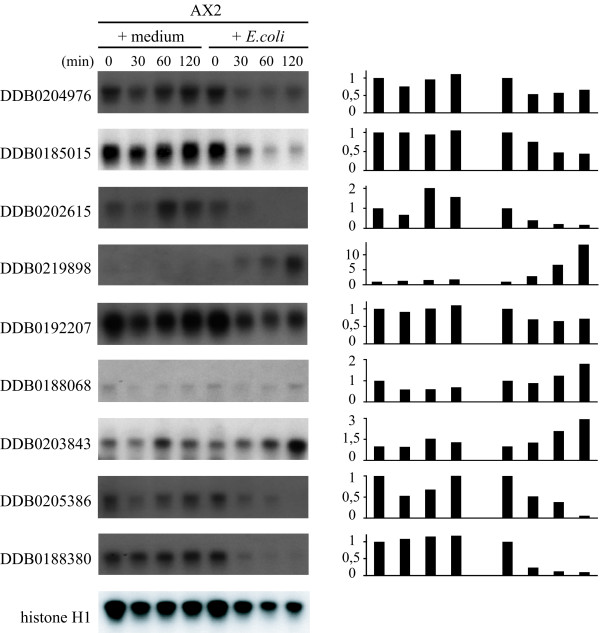
**Time course of gene expression in cells incubated in axenic medium or with *E. coli***. Exponentially growing AX2 cells were washed from axenic medium, resuspended at a concentration of 5 × 10^6 ^per ml and incubated either in axenic medium or with a 1000-fold excess of *E. coli *B/r in Soerensen phosphate buffer. At the time indicated, cells were washed and the RNA extracted with TRIzol as described in *Methods*. After agarose electrophoresis, the Northern blots were hybridized with the DNA probes indicated on the left. *(Right) *The intensity of the bands in Northern blots was calculated using ImageJ and normalized to the value of the histone *H1 *gene as internal control. The normalized values at each time point were divided for the values at time 0 and expressed as fold increase or decrease, setting the 0-point to 1.

to investigate the transcriptional response to phagocytosis, axenically growing AX2 cells were washed from medium and incubated with 1000-fold excess of *E. coli B/r *under shaking. As control, washed cells were incubated in axenic medium. After a 2 hour incubation, total RNA was extracted and used for DNA microarray analysis. The two-hour time point was chosen, because in Northern blots with unselected genes it was evident that although subtle changes in gene expression could be sometimes detected after 30 min of incubation with bacteria, accumulation or disappearance of RNA species was more consistent after 2 hours (Figure 1). On the other hand a 2-h incubation is not yet sufficient for complete adaptation of axenic cells to the new growth condition, as inferred from the fact that the first round of cell division, upon transferring axenic cells to bacteria, usually occurs after 4, sometimes 6 hours.

#### (2) Comparison B

RNA was also extracted from cells growing exponentially on bacteria or in axenic medium, thus representing a sort of "steady state", as far as growth is concerned, under both conditions. Although transcriptional changes due to phagocytosis cannot be excluded, it is more likely that they reflect metabolic adaptation to post-uptake processing of different nutrients.

#### (3) Comparison C

As additional control, AX2 cells incubated for 2 hours with bacteria were compared with cells growing exponentially on bacteria. This comparison helps in distinguishing genes adapting earlier or later to growth on bacteria. Cross-comparison with the other two experimental conditions will allow restricting the number of genes whose expression is likely regulated by phagocytic stimuli or by a combination of phagocytic stimuli and differential growth requirements. In addition, comparing the results of the three experimental set-ups will help in discriminating effects on gene expression induced by the experimental manipulation (centrifuging, washing and resuspending cells in different media).

#### (4) Comparison D

A supplemental comparison was also made between cells of the wild type strain V12M2 and AX2, both in exponential growth on *E. coli*. As mentioned in the introduction, wild type strains are strictly dependent on phagocytosis for growth, and they are more efficient phagocytes than the axenic strains. Although strain differences in gene expression have to be taken into account, as the parental wild type strain of AX2 is NC4 and the genome of V12M2 has not been sequenced, we hypothesized that this comparison may help in corroborating the results obtained with the three comparisons mentioned above.

Two separate experiments were done, the collected RNA was labelled and the resulting samples hybridized with an array covering approximately 8500 genes non-redundantly.

### Major changes in gene expression are induced early upon shifting from axenic medium to bacterial culture

**Figure 2:**
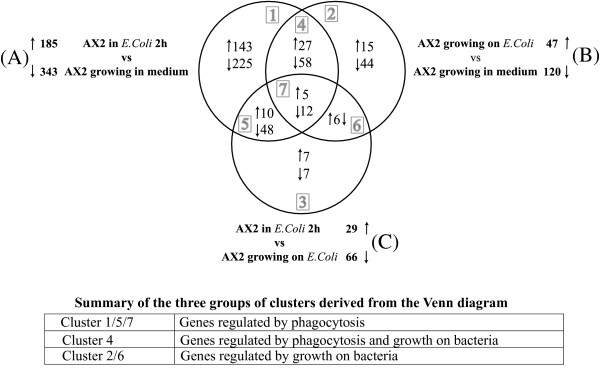
**Venn diagram of differentially regulated genes**. The total number of up- and down-regulated genes in each comparison and the numbers of differentially regulated genes in the various clusters of the three comparisons are shown in black. Arrows indicate up- or down-regulated genes. The seven possible clusters of the diagram (grey numbers) can be grouped in three larger clusters (table below): cluster 1/5/7 includes genes regulated by phagocytosis, cluster 4 genes regulated by phagocytosis and growth on bacteria, and cluster 2/6 genes regulated by growth on bacteria. "Phagocytosis" means all stimuli that may arise from bacterial binding to the membrane, particle uptake, phagosome intracellular traffic; "growth on bacteria" means stimuli arising from metabolic adaptation to the different nutrients in bacteria vs. axenic medium. See text for details.

Figure 2 summarizes the number of genes differentially expressed in each of the binary combinations that were compared. The largest variation was found in AX2 cells incubated for 2 hours with bacteria compared to axenic medium *(comparison A)*, with a total of 528 genes showing significant changes. The lowest variation (95 genes) was for cells incubated for 2 hours with bacteria compared to cells in exponential growth on bacteria *(comparison C)*. In all three comparisons, the majority of the genes were down-regulated, with a slightly lower percentage of down-regulated genes in combination A. Thus it appears that a higher number of genes is more highly expressed during growth in axenic medium than growth on bacteria, and more mRNAs are enriched in the bacterially grown cells in exponential phase than after a two hour incubation in the same food source. In addition, the finding that in comparison C only a minority of genes underwent changes indicates that: (1) changes in gene expression due to phagocytosis occurs mostly within the first two hours of incubation with bacteria, and (2) the dramatic changes observed in comparison A are not due to the experimental manipulation of the cells, but are induced by phagocytosis and/or transition to growth in bacterial culture.

A total of 513 and 520 up- and down-regulated genes, respectively, was found in comparison D (V12M2 strain compared to AX2, both growing on bacteria). These high numbers may be due to strain differences, as the wild type parental strain of AX2 is NC4, as mentioned above. They reflect, however, also changes due to the strong selection to which axenic strains have been exposed to for growing in artificial medium.

**Figure 3:**
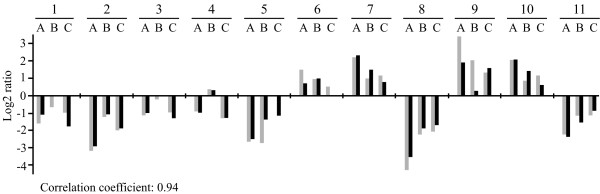
**Verification of gene expression profiles by Northern blots**. Eleven genes were selected randomly to compare their expression profiles obtained with DNA microarray with expression in Northern blots. The intensity of the bands in Northern blots was calculated using ImageJ and normalized to the value of the histone H1 gene as internal control. The log ratio of expression values in comparisons A, B and C is shown in grey for microarray and black for Northern blots. The correlation coefficient was calculated using the Excel function. Numbers on top indicate the probed genes: 1, *act15 *(DDB0185015); 2, *sevA *(DDB0188380); 3, *arcB *(DDB0204976); 4, *coaA *(DDB0192207); 5, *talA *(DDB0219577); 6, *eIF6 *(DDB0203843); 7, *gadB *(DDB0188068); 8, *aclyB *(DDB0205386); 9, (DDB0219898); 10, *rabR *(DDB0168590); 11, *nramp1 *(DDB0202615).

The microarray data were confirmed by checking the expression profiles of 11 genes by Northern blot analysis, using the gene encoding histone 1 as internal control. The results were comparable with the microarray data for most of the genes and combinations tested and the correlation coefficient was very high (Figure 3).

### Identifying clusters of genes regulated by phagocytosis or growth on bacteria

The differentially expressed genes in comparisons A-C were analyzed using the Venn diagram, to single out common and comparison-specific genes (Figure 2). Genes could be classified into all seven possible categories *(grey numbers)*. We grouped these into larger clusters according to their biological significance, as discussed below.

Seventeen genes were differentially expressed in each of the three comparisons *(cluster 7)*, while 85 and 58 differentially expressed genes were shared by comparisons A-B *(cluster 4) *or A-C *(cluster 5)*, respectively. In common to comparisons B and C were only 6 differentially expressed genes *(cluster 6)*. Interestingly, these 6 genes are down in comparison B, but up in comparison C ("up" and "down" as defined in Table 1). A likely explanation is that they are overexpressed in cells growing axenically and down-regulated during bacterial growth, but that a 2-h incubation with bacteria is not yet sufficient for complete down-regulation. If this assumption is correct, these genes, together with the genes in cluster 2, represent genes regulated mainly by the different growth conditions, meaning effects caused by the different nutrients involved *(cluster 2/6: genes regulated by growth on bacteria)*. This assumption would also explain why these genes do not undergo detectable changes in comparison A.

Similarly, assuming that comparison C represents a transient point toward the steady-state growth on bacteria, the gene clusters in common between A and C *(cluster 5)*, and among A, B, and C *(cluster 7)*, contain genes regulated by phagocytic stimuli, which are far from equilibrium in comparison C after the 2-h incubation with bacteria. In contrast, one has to assume that this equilibrium has been reached for the remaining genes of comparison A (included in *clusters 1 *and *4*) that are not detected in comparison C. Cluster 4 would then contain genes that are regulated early by phagocytic stimuli, but maintain their level of expression during steady state growth on bacteria compared to axenic growth. Together with cluster 7, cluster 4 may represent a more robust set of genes regulated by phagocytosis, though stabilizing effects of the different growth conditions for this cluster cannot be excluded. Finally, the genes differentially expressed in cluster 1 represent also an early adaptational response to phagocytosis, but biphasic: an earlier higher variation upon initial incubation with bacteria would be followed by average expression as cells adapt to growth on bacteria *(comparison B)*. Additional positive effects of the different growth conditions are excluded for these genes, otherwise they would fall in cluster 4. Potential "starving" effects, due to the cells being washed from axenic medium and resuspended on bacteria, cannot be excluded but they are unlikely, because similar effects should affect their expression in comparison C. Based on these considerations, cluster 1/5/7 are assumed to be mainly regulated by phagocytic stimuli, cluster 4 by phagocytosis and growth on bacteria, and cluster 2/6 by growth on bacteria (compared to axenic growth). A list of the genes included in these three different clusters (*cluster 1/5/7*, regulated by phagocytosis; *cluster 4*, regulated by phagocytosis and growth conditions; *cluster 2/6*, regulated by growth on bacteria) is shown in the Additional Files 1, 2, 3.

### Functional characterization of differentially regulated genes

**Figure 4:**
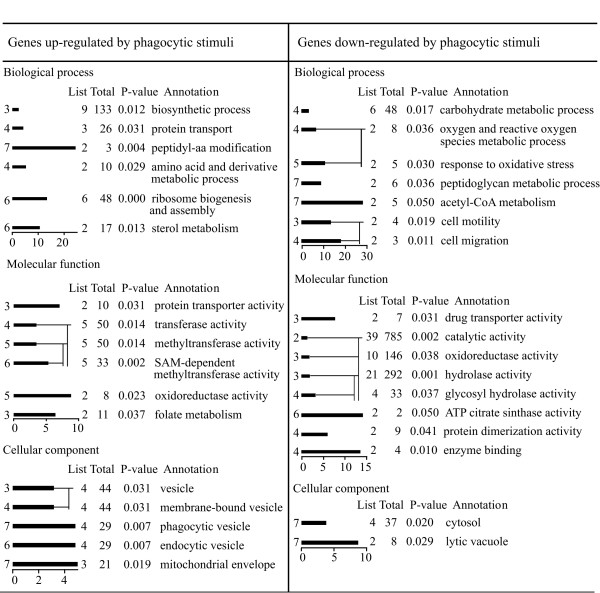
**Selection of enriched GO terms in cluster 1/5/7 (genes regulated by phagocytic stimuli)**. The GOAT programme [33] was used to calculate the enrichment (abscissa) and statistical significance (P-value) of every GO term (GO-level in the ordinate and GO-annotation on the right). The observed number of genes in a specific category (List) and the number of genes that might appear in the same category in case of random selection from the same reference list (Total) are shown. Due to redundancy in GO-terms, only a selection of enriched terms is shown.

**Figure 5:**
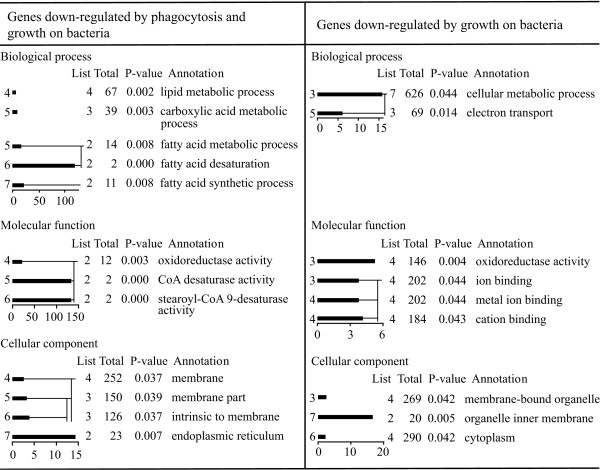
**Selections of enriched GO terms in cluster 4 and cluster 2/6**. In cluster 4 (genes regulated by phagocytosis and growth on bacteria) and cluster 2/6 (genes regulated by growth on bacteria) the GO terms resulted enriched only for down-regulated genes. See legend to Figure 4 for experimental conditions and explanations.

In order to obtain functional profiles of the differentially regulated genes, the three gene clusters identified above were subjected to Gene Ontology analysis using the GOAT programme [33]. The Gene Ontology project provides a categorization system to sort out genes in three categories: molecular functions, biological processes and cellular localization, each with several subcategories [34]. Given a gene list and a reference gene list, the GOAT programme calculates enrichment and statistical significance of every Gene Ontology term, by comparing the observed number of genes in a given category with the number of genes that might appear in the same category if a selection performed from the same reference list were completely random [33]. As reference we used the DNA microarray gene list and the results are shown in Figures 4 and 5.

It is worthwhile reminding that the GO annotations are suggestive of putative, rather than proven functions, as only a minority of *Dictyostelium *genes have been experimentally analyzed. In addition, bioinformatic tools, such as GOAT, depend heavily for their predictions on the numbers of annotated genes, their correct annotation and correct linkage to GO terms, parameters which are constantly changing. Thus, the results that follow have to be considered as preliminary and purely indicative of potential functions that need further validation by other means.

For the up-regulated genes in cluster 1/5/7 (genes regulated by phagocytosis), enriched GO terms for biological process and molecular function were aminoacid biosynthesis, peptidyl-aminoacid modification, protein transport, ribosomal protein biosynthesis and sterol metabolism. Interestingly, enrichment in protein transport is due to genes putatively encoding proteins of the mitochondrial import inner membrane translocase system (Additional File 4). Among the genes involved in peptidyl-aminoacid modification are genes encoding S-adenosylmethionine-dependent methyltransferase activity and one gene coding for formylmethionine deformylase. The latter may suggest increased activity in the hydrolysis of bacterial proteins.

Enriched GO terms for cellular components were mitochondrial envelope and phagocytic vesicle. Mitochondrial envelope was singled out because of the same translocases that had led to enrichment of protein transport. Phagocytic vesicle was correlated with expression of four genes (Additional File 4). The encoded putative proteins were recently found in a proteomic study to be enriched in phagosomes [35], which justifies their GO assignment to phagocytic vesicle. The finding that they are up-regulated in the present microarray study in response to phagocytic stimuli, and that are detected by the GOAT programme, makes these genes interesting candidates for further investigation.

The down-regulated genes in cluster 1/5/7 (genes regulated by phagocytosis) led to enrichment of GO terms linked to carbohydrate and peptidoglycan metabolism, glycosyl hydrolase activity, response to oxidative stress, drug transporter activity and cell motility (Figure 4 and Additional file 4). Among the genes responsible for these activities are genes encoding putative glycosidases, lysozymes, the ATP citrate lyase, acetylcholinesterase, lipases, ABC transporters. Calcineurin-like phosphoesterases, acid phosphatase, tyrosine-specific protein phosphatase are also proteins which contribute to these GO terms (see Additional File 4).

Cluster 1/5/7 (genes regulated by phagocytosis) is thus characterized by up-regulation of genes involved in translation, sterol biosynthesis and mitochondrial biogenesis and down-regulation of genes mainly involved in carbohydrate, peptidoglycan and lipid metabolism.

GOAT analysis of the genes regulated by phagocytosis and growth conditions (cluster 4) and the genes regulated by growth on bacteria (cluster 2/6) led to enrichment of GO terms for down-regulated genes only, for cluster 4 specifically for fatty acid metabolism, in particular putative stearoyl-CoA 9 desaturase activity (Figure 5 and Additional File 5). For cluster 2/6, GO enrichment was found for activities involving genes such as putative lipooxygenase, α-mannosidase, the extracellular phosphodiesterase precursor and cytochrome c oxidase subunits I and II.

### Manual annotation of differentially regulated genes

**Figure 6:**
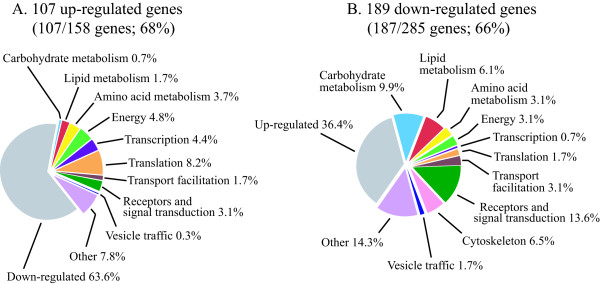
**Manual annotation of categories for cluster 1/5/7 (genes regulated by phagocytic stimuli)**. The percentage of manually annotated genes was calculated for up- or down-regulated genes (A and B, respectively). The annotated genes were categorized and the percentage of genes in each category was calculated, taking as 100% the total number of annotated genes in the cluster. Using the total number of genes as reference shows more clearly the differences in percentage within up- and down-regulated categories.

**Figure 7:**
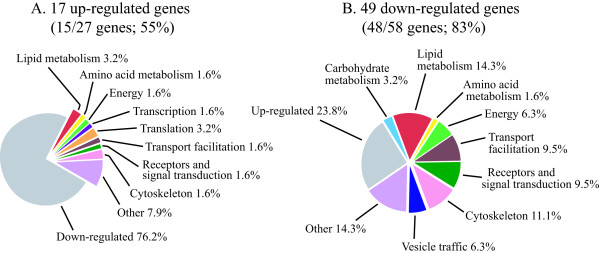
**Manual annotation of categories for cluster 4 (genes regulated by phagocytosis and growth on bacteria)**. See legend to Figure 6.

**Figure 8:**
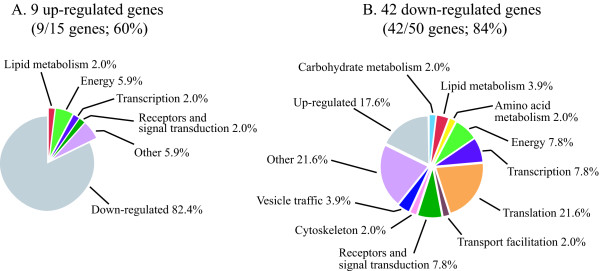
**Manual annotation of categories for cluster 2/6 (genes regulated by growth on bacteria)**. See legend to Figure 6.

As mentioned, a shortcoming of applying bioinformatic programmes such as GOAT is that they depend on progress in correct gene annotation and categorization. In contrast to characterized genes, particularly those involved in basic metabolisms, novel or less frequent genes are often either not annotated or not categorized properly, due to lack of functional or structural information. Evidence for the limitation of GOAT is provided by the above-mentioned identification of four genes as residing in phagocytic vesicles, although, as will be mentioned further below, manual annotation of the microarray data detected 25 protein products in common with the 179 proteins identified in the phagosomal proteomic profile. We therefore annotated manually the three sets of genes comprising the clusters 1/5/7, 2/6 and 4, using the latest information in dictyBase [36] and incorporating results from homology search against protein and domain databases (refseq_proteins, swissprot proteins, protein databank proteins). For each cluster, the genes were organized according to the categorization scheme for *D. discoideum *that is based on the yeast classification scheme [37]. The percentage of up- or down-regulated genes belonging to a given category for each group is shown in Figures 6, 7, 8.

#### -Cluster 1/5/7 (genes regulated by phagocytic stimuli)

In the case of genes regulated by phagocytic stimuli (cluster 1/5/7), a total of 294 genes out of 443 (66%) could be annotated. Of these 1/3 was up- and 2/3 down-regulated. Categories such as transcription and protein synthesis were up-regulated. For transcription, 13 genes were up- and only 2 down-regulated, namely the MADS-box transcription factor *srfC *[38] and the putative transcription factor *wrky1*. Similarly for protein synthesis, which includes also genes encoding ribosomal proteins, 24 genes were over- and only 5 under-expressed (see Additional File 1). Approximately equal number of genes was found for categories such as aminoacid metabolism, stress response and adhesion (Figure 6). Although up-regulated genes involved in energy production were only slightly enriched, 6 genes encoding putative proteins of the translocation machinery across the mitochondrial membrane were over-, and only 1 under-expressed (see Additional File 1), which suggests that increased mitochondrial biogenesis is an early response to phagocytic stimuli, in agreement with the GOAT data.

Genes for carbohydrate, lipid metabolism, proteolysis, signal transduction, cytoskeleton, vesicle traffic were for the vast majority down-regulated. In the case of cytoskeleton, all 19 genes annotated were down-regulated (see Additional File 1). Vesicle traffic and transport facilitation is represented with 20 genes, including 5 ABC transporters [39], which are all down with exception of *abcA2*. Two genes for putative Rab proteins are also worth mentioning, *rabR *(DDB0168590) and *rabQ *(DDB0190014), which are up and down, respectively (see Additional File 1).

In the case of signal transduction, 40 genes, including putative membrane receptors, GEF's and GAP's, phospholipases B and calcineurin-like phosphatases are down-regulated, with the notable exception of the genes encoding a myotubularin 2-like protein (*mtmr2*, DDB0188079), the phosphatidylinositol phosphatase (*plip*, DDB0168924) and a putative STE protein kinase (*mkcG*, DDB0190837). The *rta1 *gene (DDB0168536), encoding a potential receptor, is also over-expressed about 2.5 fold. Interestingly, 4 of 7 genes in the whole *D. discoideum *genome that encode potential phospholipases B are included in this cluster and are all down-regulated (see Additional File 1).

#### -Cluster 4 (genes regulated by phagocytosis and growth on bacteria)

A total of 63 genes could be annotated in cluster 4, amounting to 74% of the genes in this cluster, which is characterized by a net prevalence of categories enriched among the genes down-regulated (Figure 7). The hypothesis that cluster 4 is regulated by both phagocytic stimuli and growth on bacteria is substantiated by the general tendency of carbohydrate and lipid metabolism, energy, transport facilitation, cytoskeleton and signal transduction to be down-, and transcription, translation and stress response up-regulated in this cluster, following the same trend observed for cluster 1/5/7 (see Additional File 2). However, 5 genes only account for the last three categories in cluster 4 against 43 in cluster 1/5/7 (genes regulated by phagocytosis). Interestingly, no genes required for mitochondrial transport system or electron transport and respiration are present in cluster 4, in contrast to cluster 1/5/7, where they are mostly up-regulated. This confirms that changes in mitochondrial build-up are induced early in response to phagocytic stimuli. Similarly to cluster 1/5/7 (genes regulated by phagocytosis), 7 genes encoding cytoskeletal proteins are down in cluster 4. Notable exceptions is the gene encoding ponticulin A (*ponA*, DDB0192061), which is up-regulated by about 3.5 fold (see Additional File 2).

#### -Cluster 2/6 (genes regulated by growth on bacteria)

A total of 51 genes, out of 65 (78%), were annotated in cluster 2/6. Only 9 annotated genes are overexpressed in this cluster, distributed in 5 categories. The very low number and the dispersion in 5 categories preclude any conclusion about these data. Carbohydrate and fatty acid metabolism, vesicle traffic, transport facilitation, cytoskeleton and signal transduction are down-regulated in cluster 2/6 (Figure 8), as previously shown for cluster 1/5/7 and cluster 4, confirming that most of these changes can be considered adaptational responses to the different growth conditions, independently of whether they are early or late responses.

## Discussion

*Dictyostelium *cells exploit phagocytosis to get nutrients and as a defense mechanism against pathogens. We have tried to identify genes regulated by phagocytosis or growth on bacteria, by comparing gene expression changes under different conditions: in axenically growing cells, which were washed and exposed to bacteria for two hours compared to cells either in axenic medium or growing on bacteria; in addition, gene expression was studied during exponential growth on bacteria versus axenic medium. The last comparison would reveal changes in gene expression at a steady state of differential growth. Although these changes may be also due to phagocytosis, it is more likely that they reflect metabolic adaptation to post-uptake processing of different nutrients. Cross-comparison has allowed restricting the number of genes whose expression is likely regulated by phagocytic stimuli or by a combination of phagocytic stimuli and differential growth requirements.

The 2 h time-point of incubation with bacteria is a good compromise to assess effects of phagocytosis, as evidenced by the time course of gene expression in Figure 1 and the results of the gene expression profile in cells incubated for 2 hours with bacteria compared to cells growing exponentially with bacteria (comparison C): 95 genes are still far from the equilibrium that one would expect if cells had completely adapted to growth on bacteria within 2 hours. This analysis has led to identification of two classes of genes, induced by phagocytosis: an early and a late adapting one, including 453 and 73 genes, respectively.

We also compared gene expression in the wild type strain V12M2, which grows strictly on bacteria, with AX2 growing on bacteria. V12M2 is not the parental strain of AX2, yet despite strain differences several genes undergoing changes in cluster 1/5/7 (genes regulated by phagocytosis) or cluster 4 (genes regulated by phagocytosis and growth on bacteria) were present in this comparison, and their trend of regulation was in many cases strikingly similar, though the absolute values of enrichment could vary. This probably reflects the fact that the wild type V12M2 strain is a more efficient phagocyte than AX2, and supports the idea that the results emerging from this comparison can be used as additional internal control for gene categories or for specific genes of interest. With very few exceptions that are mentioned in the discussion below, we have used these data solely as corroborating control. A complete list of the regulated genes in comparison D has been however deposited and is available at ArrayExpress.

A first conclusion that can be drawn from this study is that axenically growing cells require a larger number of genes to be more highly expressed than bacterially growing cells: by comparing both cells incubated for 2 hours with, or in exponential growth on bacteria with cells growing axenically, about 64 to 77 % of the genes undergoing significant changes are down-regulated in the presence of bacteria. GOAT analysis, has shown significant down-regulation of genes involved in carbohydrate and lipid metabolism, proteolysis, transport facilitation, cytoskeleton and signal transduction, and up-regulation of genes involved in stress response, transcription, protein synthesis and mitochondrial biogenesis. These results are consistent with a rapid metabolic shift from a carbohydrate-rich, but probably less equilibrated, artificial medium to the natural food source of *Dictyostelium *cells.

### -Lipid metabolism: a role for sterols in phagocytosis?

There were some interesting exceptions, detected by GOAT analysis and confirmed by manual annotation, such as up-regulation of sterol metabolism in the otherwise down-regulated category of phospholipid, fatty acid and isoprenoid metabolism in both cluster 1/5/7 (genes regulated by phagocytosis) and cluster 4 (genes regulated by phagocytosis and growth on bacteria). Taken together, 78% of genes involved in fatty acid and phospholipid metabolism are downregulated, a few very strongly, such as two genes (*aclyA*, DDB0205389, *aclyB*, DDB0205386) coding for the ATP citrate lyase, and *pks16 *(DDB0167446), encoding a putative fatty acid synthase, which are down-regulated 16 to 20-fold. By contrast, sterol metabolism is a GO term enriched in cluster 1/5/7, due to up-regulation of the gene *sqle *(DDB0192021) encoding the squalene monooxygenase, a key enzyme for the synthesis of squalene-2,3-epoxide, the precursor of lanosterol.

Interestingly, up-regulated in cluster 1/5/7 are also the gene *acact *(DDB0168409) encoding the acetyl-CoA acetyltransferase, which catalyzes the formation of aceto-acetyl-CoA, the first compound in the pathway leading to squalene, and the gene DDB0190243, encoding a putative sterol isomerase. The gene *ggps1 *(DDB0192027) coding for geranyl-geranyl pyrophosphate synthase, involved in isoprenoid metabolism, is up-regulated in cluster 2/6 (gene regulated by growth on bacteria). The product of this enzyme, the geranyl-geranyl-pyrophosphate, is a precursor of squalene. Down-regulated among the genes regulated by phagocytosis (cluster 1/5/7) is *hgsA *(DDB0219349), encoding the 3-hydroxy-3-methylglutaryl-CoA-synthase, which leads to both squalene synthesis and ketone bodies. The genes described above were similarly regulated in V12M2 when compared with AX2 growing on bacteria, with the notable exception of *hgsA* (DDB0219349), which is almost unchanged (*data not shown*). In addition, two genes encoding a putative steroid reductase (DDB0189425), which is required for ergosterol biosynthesis, and steroid isomerase (*sre1*, DDB0204248) are overexpressed 2-fold in V12M2. Thus it appears that phagocytosis and growth on bacteria are accompanied by up-regulation of various genes involved in sterol biosynthesis and down-regulation of genes controlling fatty acid and phospholipid metabolism.

Sterol is enriched in the membrane of *Dictyostelium *phagosomes, digestive vacuoles and autophagic vacuoles [40]. We have found no data in the literature concerning differences in sterol composition between membranes of axenically or bacterially growing cells, but the present results suggests that a lipidomic comparison of phagosomes with macropinosomes is due, and could reveal interesting differences. Sterols are important components of lipids rafts and their role in regulating phagocytosis is slowly emerging. Cholesterol rich membrane rafts have been shown to mediate phagocytosis of *Mycobacterium*, *Pseudomonas *or *Leishmania *species in macrophages [41-44]. De Chastellier and Thilo [45] have recently shown that cholesterol depletion weakens the close apposition of phagosomal membrane and the membrane of mycobacteria, interfering with phagosome maturation.

### -Carbohydrate metabolism and phagocytosis

Carbohydrate metabolism is mostly down-regulated in cluster 1/5/7 (genes regulated by phagocytosis), with 29 under-expressed genes against only 2 overexpressed, amounting to 10.5 % of the annotated genes undergoing changes in this cluster. The same category is represented with only 2 genes in cluster 4 (genes regulated by phagocytosis and growth on bacteria) and 1 gene in clusters 2/6 (genes regulated by growth on bacteria), respectively, suggesting that most changes in carbohydrate metabolism occur very early in response to phagocytic stimuli. Many of the genes in this category encode glycosidases, such as the lysosomal α-mannosidase precursor (*manA*, DDB0184287) [46], acid α-glucosidase (*gaa*, DDB0190556), β-mannosidase (*manH*, DDB0217722), α-amylases (*gtr2*, DDB0169506, *amyA*, DDB0204542) [47] and putative glycosyl hydrolases (DDB0167078, DDB0190788), in addition to some dehydrogenases. They are all down-regulated, in agreement with previous results showing that cells growing on bacteria do not express glycosidases, which are instead expressed in cells growing axenically [48,49]. It is likely that the more luxuriant condition of bacteria as source of food, in contrast to the artificial carbohydrate-rich axenic medium, does not require high expression of these enzymes.

Interestingly, the two up-regulated genes in the carbohydrate category encode a C-type (*lyC2*, DDB0189256) and a T4-type (*lyT4-2*, DDB0167824) lysozyme, whereas four members of the lysozyme ALY family (*alyA*, DDB0167491, *alyB*, DDB0167489, *alyC*, DDB0167490 and *alyD-2*, DDB0217279), were downregulated. The ALY (Amoeba LYsozymes) proteins represent a novel lysozyme family, which includes four members and is not related to any known lysozyme family [50]. The *alyA *product has been shown to display lysozyme activity *in vitro *as well as antibacterial activity, but against Gram-positive bacteria, whereas Gram-negative bacteria, such as *E. coli*, are unaffected by the enzyme [50]. The finding that these genes are down-regulated in cells incubated with *E. coli *suggests that the *aly *genes are not required for *E. coli *degradation, whereas genes encoding the C- and T4-type lysozymes, which are up-regulated 2 to 5-fold, could be important for *E. coli *peptidoglycan hydrolysis.

### -Aminoacid metabolism and phagocytosis

**Figure 9:**
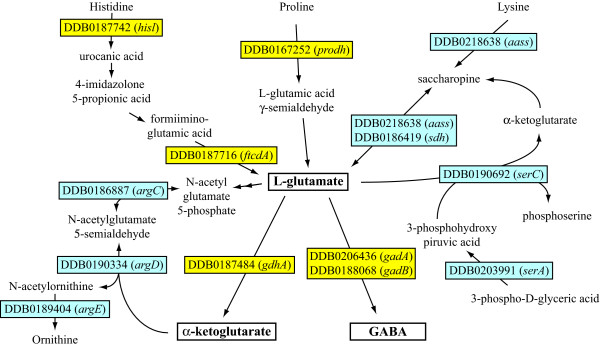
**Regulation of glutamate metabolic pathway by phagocytic stimuli inferred by the gene expression profile**. Twenty genes involved in aminoacid metabolism are detected in the DNA microarray. Of these, 14 are directly or indirectly linked to glutamate metabolism, as shown in the Figure. Over- and under-expressed genes are shown in yellow and grey, respectively. *GadA *(DDB0206436) and *gadB *(DDB0188068), encode the glutamate decarboxylase A and B [51], respectively and are strongly over-expressed. Up-regulated are also a putative proline dehydrogenase (*prodhA*, DDB0167252), which catalyzes the formation of L-glutamic acid γ-semialdehyde, the histidine ammonia lyase (*hisl*, DDB0187742) and the glutamic acid formiiminotransferase (*ftcdA*, DDB0187716), which are both involved in the major pathway for converting histidine to glutamic acid. Up-regulated is also the gene for the 10-formyltetrahydrofolate synthetase (*fths*, DDB0188868), which generates a potential substrate for the formiimino group released by the formiiminotransferase. Slightly up-regulated is also the gene for the glutamate dehydrogenase (*gdhA*, DDB0187484), which leads to α-ketoglutarate formation from L-glutamic acid. Downregulated are seven genes encoding putative enzymes involved in the conversion of L-glutamic acid to, respectively, L-ornithine (*argC*, DDB0186887, *argD*, DDB0190334 and *argE*, DDB0189404), serine (*serA*, DDB0203991 and *serC*, DDB0190692) or lysine (*aass*, DDB0218638 and *sdh*, DDB0186419). In the assumption that the regulation of all these genes is reflected at enzymatic level, a preferential accumulation of glutamate and its conversion to GABA and α-ketoglutarate could be inferred.

Aminoacid acid metabolism in the cluster 1/5/7 (genes regulated by phagocytosis) is represented with 20 genes, whereas only 2 genes are present in cluster 4 (genes regulated by phagocytosis and growth on bacteria) and 1 in cluster 2/6 (genes regulated by growth on bacteria). Two genes, *gadA *(DDB0206436) and *gadB *(DDB0188068), encoding the glutamate decarboxylase A and B [51], respectively, are strongly over-expressed. Eleven other genes are directly or indirectly linked to glutamate metabolism, as summarized in Figure 9. The coordinated up- and down-regulation of all these genes, if reflected at the level of enzymatic activities, suggests accumulation of L-glutamic acid, which through the activity of the glutamate decarboxylases A *(gadA) *and B *(gadB) *or the glutamic acid dehydrogenase *(gdhA) *will lead to an increase in GABA (γ-aminobutyric acid) or conversion to α-ketoglutarate, respectively (Figure 9). Glutamate, as precursor of α-ketoglutarate and, *via *the GABA shunt, of succinate may represent the final step in the catabolism of aminoacids and the major source of energy in bacterially growing cells. GABA has been shown to regulate late differentiation in *Dictyostelium via *binding to the metabotropic-like receptor GrlE [51]. Whether GABA or glutamate are released during phagocytosis and thus may act as autocrine signals to regulate the phagocytic response is unknown, but this is a possibility to be tested.

### -Transcription, translation, mitochondrial biogenesis and stress response

Genes involved in transcription and translation, ribosomal and mitochondrial biogenesis and stress response appear to be up-regulated by phagocytosis. This is not surprising, as cells grow on bacteria more rapidly than in axenic medium, therefore it is likely that phagocytosis triggers gene expression changes leading to increased ribosomal and mitochondrial biogenesis as well as protein synthesis. A total of six genes encoding heat shock proteins (HSP), including HSP 70 family protein (*hspN*, DDB0186670) are up-regulated among genes induced by phagocytosis (cluster 1/5/7) and genes induced by phagocytosis and growth (cluster 4). Two of them (*grpE*, DDB0185728 and *cpn10A*, DDB0187651) encode chaperonins involved in mitochondrial transport and their up-regulation could be linked to increased mitochondrial biogenesis. HSP's have been shown to modulate inflammatory response and phagocytosis in macrophages, though their mechanism of action remains unknown [52,53].

Putative genes encoding transcription factors were detected only in cluster 1/5/7 (genes regulated by phagocytosis). A homeodomain-containing protein (*hbx3*, DDB0219656), and the transcription factor *jcdG *(DDB0187500) of the jumonji family are up-regulated, the MADS-box transcription factor *srfC *(DDB0202276) [38], and a putative WKRY transcription factor (*wrky1*, DDB0217613) down-regulated. Their function in *Dictyostelium *is unknown, but they are potential candidates for regulating early gene expression in response to phagocytic stimuli. WKRY transcription factors, in particular, have been shown to be positive and negative regulators of pathogen defense pathways in plants [54].

### -Intracellular traffic and cytoskeleton

**Figure 10:**
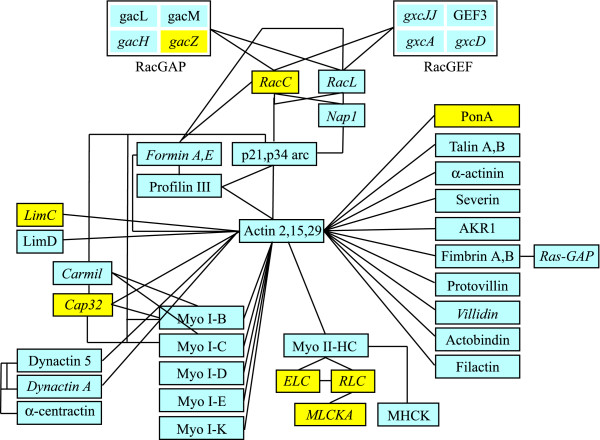
**Regulation of genes encoding actin cytoskeleton proteins by phagocytosis and growth on bacteria**. The figure shows the protein products of genes for actin cytoskeletal proteins and their small G protein regulators, which are detected in the microarray, with their interactions as known from literature. Over- and under-expressed genes are shown in yellow or grey, respectively. In italics are indicated genes, which are detected in the comparison V12M2 cells vs. AX2 growing on bacteria. The vast majority of these genes is down-regulated. See text for comments. For the DDB ID numbers of the genes see Additional Files 1, 2, 3.

The *Dictyostelium *genome harbours 58 genes encoding Rab proteins, as well as several SNARE's and other proteins of the vesicular traffic. They are strongly underrepresented among genes regulated by phagocytosis (clusters 1/5/7) or phagocytosis and growth on bacteria (cluster 4), with only *rabR *(DDB0168590), which is up-regulated, *rabQ *(DDB0190014), a dynamin-like protein (*dymA*, DDB0204411), a beta-adaptin-like protein (*ap1b1*, DDB0204689) and a putative Vps protein (*vps13*, DDB0187116), which are all down-regulated. This suggests that most of the genes involved in intracellular vesicle traffic and fusion do not undergo changes when cells switch from macropinocytosis to phagocytosis. The 4-fold increase in *rabR *may suggest a specific role for this gene in phagosomal traffic. A *dymA*-minus mutant displays reduced fluid-phase uptake but increased phagocytosis [55], which would be consistent with the gene down-regulation observed here, suggesting a differential requirement for dynamin in endocytosis not phagocytosis, similarly to what has been described for macrophages [56]. A total of 27 genes encoding cytoskeletal proteins are represented in clusters 1/5/7 (genes regulated by phagocytosis) and cluster 4 (genes regulated by phagocytosis and growth on bacteria), and they are all down-regulated, with the notable exception of the ponticulin encoding gene *ponA *(DDB0192061). The genes encoding severin (*sevA*, DDB0188380), filactin (*fia*, DDB0185986), talin A (*talA*, DDB0219577), protovillin (*vilB*, DDB0167024) and fimbrin (*fimA*, DDB0204382) in particular are strongly down-regulated. Phagocytosis is an actin-based process, thus down-regulation of several components is surprising. On the other hand, despite many actin-binding proteins and myosin I isoforms are transiently recruited to phagosomes, only a handful of them have been shown by gene knock-out experiments to affect phagocytosis, possibly due to redundancy of the system [4,9,57-59]. Remarkably, with the exception of ponticulin, many of these genes are down-regulated also in the comparison D (V12M2 *vs*. AX2 growing on bacteria), where in addition 15 other cytoskeletal genes are underexpressed (Additional File 6 and Figure 10). The extensive down-regulation of cytoskeletal proteins may paradoxically be explained with the fact that phagocytosis is a more efficient uptake system than fluid-phase endocytosis and macropinocytosis. It is conceivable that the latter processes require more extensive membrane activities, in term of membrane ruffling and formation of small and larger pinocytic cups and vesicles, to compensate for the reduced amount and "quality" of ingested food. Specific binding of bacteria to the membrane, their larger and homogenous size and their more complete nutrient content should facilitate efficient and more "rewarding" uptake than soluble and particulate nutrients contained in axenic medium. The up-regulation of the *ponA *gene is consistent with the reported overepression of ponticulin A in cells growing on bacteria, though a *ponA *knockout is not defective in phagocytosis [60].

### -Potential membrane receptors and cell signalling pathways in phagocytosis

**Figure 11:**
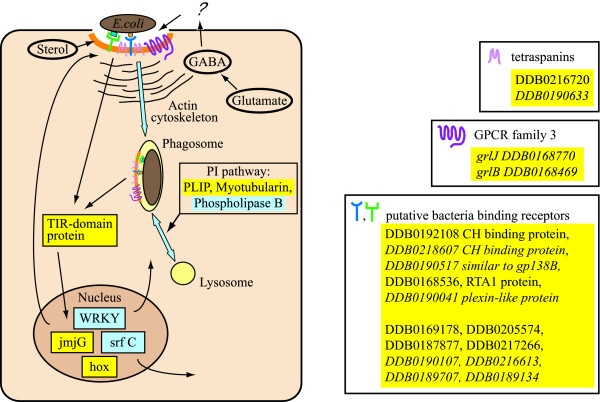
**Simplified model of regulation of differential gene expression in phagocytosis and growth on bacteria**. The model depicts some differentially regulated genes in phagocytosis and growth on bacteria, with emphasis on genes encoding putative membrane receptors, signal transducers, transcription factors and selected metabolic pathways. Over- or under-expressed genes are indicated in yellow and grey, respectively. Genes detected in the comparison V12M2 vs. AX2 growing on bacteria are indicated in italics. Phagocytosis leads to overexpression of putative membrane receptors, tetraspanins and metabotropic receptors. Receptor clustering due to bacterial binding, and to the scaffold activity of tetraspanin, may generate signalling complexes containing also GPCR's. Activation of the heterotrimeric G protein [5], putatively *via *autocrine signals or bacterial metabolites binding to GPCR's, will regulate actin reshaping in the phagocytic cup, favouring phagocytosis. We further suggest that the same signal complex at the membrane of the phagocytic cup or the engulfed phagosome may generate signals regulating gene expression, which can be mediated by a TIR-domain containing protein acting on transcription factors. Up-regulation of genes involved in sterol metabolism leads to increased production of sterols, which we suggest to be preferentially incorporated in phagocytic cups and phagosomal membrane. GABA may accumulate as catabolic product of glutamate, due to the high activity of GadA and GadB glutamate decarboxylase. Whether it is released and acts as autocrine signal is open. Regulation of phosphoinositides may regulate phagosomal maturation and fusion with lysosomes. See Additional Files 1, 2, 3 for DDB ID numbers of genes encoding the protein products indicated. The DDB ID numbers in the lower box, without protein product indication, identify genes, which are strongly stimulated by phagocytosis and encode hypothetical membrane proteins with no identifiable domains.

Cell signalling during phagocytosis is virtually unknown in *Dictyostelium*, except for the involvement of the heterotrimeric G protein and PLC in the process [1,5,35]. It was therefore of major interest to examine whether genes encoding putative membrane receptors, adhesion proteins, signal transducers and effectors were present in the microarray. As shown in Additional Files 1, 2, 3, a few such genes are detected in both cluster 1/5/7 (genes regulated by phagocytosis) and cluster 4 (genes regulated by phagocytosis and growth on bacteria). Among the up-regulated genes are worth mentioning genes encoding: a carbohydrate-binding membrane protein (DDB0192108), which is enriched about 8-fold, a gp138-similar protein (*gp138B2*, DDB0217521) [61], the seven transmembrane RTA1 protein (*rta1*, DDB0168536), putatively involved in resistance to xenobiotic stimuli, a putative TIR-like domain containing protein (*tirC*, DDB0189226), a Bystin-similar protein (*bys1*, DDB0187994) and a tetraspanin family protein (*tspC*, DDB0216720). The latter was also slightly enriched in V12M2 cells *vs*. AX2 growing on bacteria, where a second tetraspanin gene (*tspB*, DDB0190633) was up-regulated. All these proteins are potential candidates for mediating bacterial binding and membrane signals and for regulating uptake. Carbohydrate-binding receptors have been long suggested to be involved in *Dictyostelium *phagocytosis, though their identification has been elusive so far [22,62,63]. Tetraspanins are the major structural block of a class of specialized membrane microdomains and regulate lateral clustering and signalling involving cell adhesion, membrane receptor and effector proteins. Nothing is known about their function in *Dictyostelium*, but recent evidence in mammalian cells suggests their involvement also in the early steps of phagocytosis [64,65]. A similar role for the tetraspanins identified in this study is likely. TIR-like proteins, among which are Toll receptors, are major regulators of phagocytosis and pathogen-response in animals [54,66-68]. Toll-like transmembrane receptors have not been identified so far in *Dictyostelium*, but at least two genes encode cytosolic proteins with TIR-like domains, the one (*tirC*, DDB0189226) described here, and *tirA *(DDB0232375). The latter has been recently shown to be involved in an immune-like phagocyte response in *Dictyostelium *cells at later stages of development [69]. Interestingly, intracellular effectors with TIR-like domains have been recently shown to enter the nucleus and to regulate immune-like response in plants, acting on WRKY transcription factors, which negatively regulate such response [54,66,68]. It is intriguing that the *tirC *and the *wkry1 *genes are over- and under-expressed, respectively, during phagocytosis in *Dictyostelium*. It is tempting to speculate that they could interact with each other and regulate gene expression in response to phagocytic stimuli (Figure 11).

Only one gene encoding a protein kinase of the STE family (*mkcG*, DDB0190837) and three genes for phosphatases (*mtmr2*, DDB0188079, *clpeA*, DDB0185527 and *plip*, DDB0168924) were up-regulated. *Mtmr2 *encodes a protein highly similar in a domain to mammalian myotubularin-related protein 2, a dual phosphatase targeting both tyrosine and phosphoinositides [70]. The product of the *plip *gene is also a phosphatidylinositol phosphatase with PI5P as preferential substrate [71]. Interestingly, no genes encoding PI3K or PTEN, PLC, PLA2 or PLD were detected as differentially expressed in the microarray, whereas four genes encoding the phospholipase B were all down-regulated (see Additional File 3). The substrates of these phospholipases B have been shown to be the diacylglycerol-phosphocholine and -phosphoethanolamine as well as phosphatidylinositol [72]. Down-regulation of the phospholipase B encoding genes and up-regulation of *mtmr2 *and *plip *genes suggest a central role for phosphoinositides during phagocytosis. PI3K and PTEN, in contrast to PLC, are not essential for bacterial uptake [5,73] [Balest, unpublished results], but *pi3k1-2 *null mutants are defective in phago-lysosomal maturation [17,74] [Balest, unpublished results]. A phagocytosis defect has also been shown in a mutant for the inositol-5-phosphatase encoded by the *Dd5p4 *gene [75], and different phosphoinositide forms have been described being transiently activated during phagosome formation and maturation both in *Dictyostelium *and macrophages [17,75,76]. The myotubularin 2-related protein and PLIP may contribute in regulating the phosphoinositide pathway during phagosomal maturation and are thus interesting candidates for functional studies (Figure 11).

Two genes, *grlB *(DDB0168469) and *grlJ *(DDB0168770), encoding GPCR family 3 receptors, are overexpressed in V12M2 cells compared to AX2 growing on bacteria (not shown). The latter gene is also up-regulated in cluster 4 (genes regulated by growth on bacteria) AX2 cells, but below 2-fold, and so not included as significant in our analysis. Like the other members of GPCR family 3, the protein products of these genes possess a seven transmembrane domain and an extracellular domain responsible for ligand binding, with similarity to the BMP (Basic Membrane Protein) domain, initially found in outer membrane proteins of bacteria, and to the VFMT (Venus Flytrap Module) of metabotropic GPCR's [77,78]. A *grlJ-*null mutant was recently generated and found to undergo precocious development and abnormal spore differentiation [79]. Phagocytosis was not tested in the mutant. These G protein-coupled receptors could be recruited with other membrane receptors, which mediate bacterial adhesion, to form a signalling complex in sterol-and tetraspanin-regulated microdomains, as schematized in Figure 11. Receptor clustering on the site of bacterial binding was proposed long ago to explain heterotrimeric G protein activation, which in *Dictyostelium *strongly stimulates phagocytosis by favouring actin recruitment to the progressing phagocytic cup [5]. Tetraspanins have been shown to act as scaffold also for G protein-coupled receptors [80]. It is tempting to speculate that bacterial metabolites or autocrine signals, produced in response to bacterial binding, may act as local activator of GPCR's in tetraspanin-dependent signalling complexes with bacteria-binding membrane proteins. Downstream responses could be regulation of the actin cytoskeleton and of gene expression (Figure 11).

Among the several genes encoding unknown proteins some code for potential membrane receptors, as their products contain putative signal peptide, signal sequence for phospholipid anchor and/or transmembrane domains. Strongly up-regulated in cluster 1/5/7 (genes regulated by phagocytosis) are the genes DDB0219898 and DDB0168340, and in cluster 4 (genes regulated by phagocytosis and growth on bacteria) the genes DDB0217266, DDB0168342 and DDB0205574. With exception of DDB0217266, the other four genes are up-regulated also in V12M2 compared to AX2 growing on bacteria

(see Additional File 6). Worth mentioning is also the gene DDB0218607, which is over-expressed 32-fold in V12M2 cells. This gene encodes a protein with a peptidoglycan LysM domain, potentially involved in bacterial cell wall binding and/or degradation. Three other highly expressed genes in V12M2 coding for hypothetical membrane proteins are DDB0190107, DDB0216613 and DDB0190517 (see Additional File 6 and Figure 11).

### -Comparison between transcriptomic profile and the phagosomal proteome

A detailed characterization of the *Dictyostelium *phagosomal proteome was provided recently by Soldati and coworkers [35]. They resolved the time-course of over 1000 spots in purified phagosomes and identified by mass spectrometry 179 proteins. As mentioned above, four of these proteins were detected by the GOAT analysis as present among the differentially regulated genes of the microarray. While this result is interesting, as these genes encoded unknown proteins that were labelled as components of phagocytic vesicles solely because of their detection in the phagosomal proteome, it was curious that only 4 of the potentially 179 proteins of the proteomic profile were singled out by GOAT. Obviously the microarray approach detects only genes that are differentially expressed in the comparisons under study, while a proteomic characterization will encompass proteins that are residents of, or transiently recruited to the phagosome, independently of their quantitative changes during the time-course of cell incubation with particles.

Nevertheless, a manual comparison detected 20 proteins of the phagosomal proteome, in addition to the four identified by GOAT, whose genes were differentially expressed among the genes regulated by phagocytosis (cluster 1/5/7). The number increased to a total of 53 if genes differentially expressed in comparison D (V12M2 vs. AX2 growing on bacteria) were also taken in consideration. As shown in Additional File 7, these genes are distributed in several categories, and for the vast majority of them it is quite possible that the cellular localization is other than "phagosomal" in the reference databases used by GOAT. Whether these proteins are truly phagosomal or simply contaminants remains open, but it is of interest for further analysis that these proteins, and the encoding genes, are detected by both the proteomic and the microarray approaches. Among the genes expressed in V12M2 are worth mentioning genes encoding three V-H+ ATPase subunits, Rab7, calreticulin, gp24, discoidin II and gp130. The V-H+ ATPase, Rab7 and calreticulin are typical markers of phagosomal traffic [16,81-83], whereas gp24, discoidin II and gp130 are extracellular or membrane proteins that could be entrapped in the phagosome. However, knockout mutants for both gp24 and gp130 are not defective in phagocytosis [84]. Discoidins have not been involved in phagocytosis sofar, but their role could be re-evaluated, considering this result, their lectin activity and the finding that both discoidin I and discoidin II genes are strongly up-regulated in V12M2.

## Conclusion

The DNA microarray approach has pinpointed some metabolic pathways, which are differentially regulated by phagocytosis and/or growth on bacteria, and has led to identification of several interesting genes, some encoding signal transducers, transcription factors, membrane receptors and other hypothetical membrane proteins. The latter are in part orphan genes, which are conserved evolutionary, but whose function is unknown. Candidate genes can now be selected for generating null or overexpressor mutants and analyzing their phenotype in phagocytosis, growth and host-pathogen interactions. In addition, the microarray data presented here can be used for comparison studies with transcriptomes during phagocytosis of pathogenic invasive bacteria, in order to identify common and infection-specific changes in gene expression.

## Methods

### Cell strains and growth conditions

*Dictyostelium *cell strains AX2-214 and V12M2 were used. AX2 cells were grown axenically as described [21]. Wild type V12M2 cells were grown on nutrient agar plate in association with *E. coli *B/2. For growth in suspension, spores were inoculated in liquid cultures of 1 × 10^10 ^per ml of *E. coli *B/r in 0.017 M Soerensen phosphate buffer and incubated as described [85].

### Phagocytosis and growth experiments

For assaying the effects of phagocytosis on gene expression, axenically growing AX2 cells in exponential phase (3–4 × 10^6 ^per ml) were washed twice in Soerensen phosphate buffer and resuspended at a final concentration of 5 × 10^6 ^per ml either in axenic medium or in Soerensen phosphate buffer containing a 1000-fold excess of *E. coli *B/r. The cell suspension was incubated under shaking at 150 rpm on a gyratory shaker at 23 +/- 1°C (Clim-O-shaker, A. Kuehner, Birsfelden, Switzerland). After 2-h incubation, the cells were washed twice in Soerensen phosphate buffer and the pellet resuspended in TRIzol for RNA extraction.

For assaying effects of growth in axenic medium or on bacteria, AX2 spores were inoculated in axenic medium or Soerensen phosphate buffer containing 1 × 10^10 ^*E. coli *B/r per ml and the cells were harvested during exponential growth (at a cell concentration between 3 and 4 × 10^6 ^cells per ml for axenically growing cells and at 3.5 × 10^6 ^per ml for bacterially-growing cells). V12M2 cells growing in *E. coli *B/r were harvested at a concentration of 4 × 10^6 ^per ml. At the time of harvest, the extracellular bacteria were above 2 × 10^9 ^per ml in all cases.

### RNA isolation, Northern blotting and RNA quantification

RNA was extracted with TRIzol (Invitrogen, Carlsbad, CA, USA) according to manufacturer's instructions. RNA electrophoresis on denaturing agarose-formaldehyde gels and Northern blotting were done as described [25,86]. Northern blots containing RNA extracted from AX2 cells incubated for 2 hours in axenic medium or bacteria or in exponential growth on bacteria were hybridized with the following DNA probes: *act15 *(DDB0185015), *sevA *(DDB0188380), *arcB *(DDB0204976), *coaA *(DDB0192207), *talA *(DDB0219577), *eIF6 *(DDB0203843), *nramp1 *(DDB0202615), *aclyB *(DDB0205386), *gadB *(DDB0188068), *rabR *(DDB0168590), *histone H1 *(DDB0191459) and DDB0219898. The latter five probes were purified from the DNA clones VSF546, VSH267, VSG476, SLF687 and SSC146, respectively, by using restriction enzymes *KpnI *and *SacI*. These clones were generously supplied from Dr. H. Urushihara [37,87]. Quantification of the RNA bands was calculated by measuring the optical density with ImageJ and normalizing the values for the value of histone H1.

### RNA labelling, hybridization and microarray analysis

For microarray analysis, two independent RNA samples were prepared of each condition to be examined. For the comparisons of axenically grown cells with cells incubated with bacteria for 2 hours, and of V12M2 cells with AX2 cells growing exponentially in bacteria, four hybridizations were made: two technical replicates of each biological replicate, using both dye orientations. One of the axenic versus 2-hr in bacteria arrays was excluded from the analysis because of unusually uneven hybridization. For the other two comparisons, a single technical replicate was carried out for each biological replicate, with one in each dye orientation. Aliquots of 25 μg from each RNA sample were primed with anchored oligo(dT), labeled separately with Cy3 and Cy5 by direct incorporation of the dye-dCTP conjugate (GE Healthcare) in a reverse transcription reaction (Superscript III, Invitrogen Carlsbad, CA, USA), and co-hybridized to DNA microarrays comprising 9247 PCR products designed to be specific to 8579 predicted *Dictyostelium discoideum *genes, plus controls, printed in duplicate.

Arrays were scanned using an Axon Instruments Genepix 4000B scanner and the resulting images quantified (Genepix 3.0, Axon Instruments) and analyzed using the Bioconductor package LIMMA [88,89]. Background fluorescence was subtracted [90], and the resulting log ratios normalized by the print-tip loess normalization. The overall log ratio for each gene was obtained using linear model fitting (least squares) and the significance of the apparent differential expression was assessed by a Bayesian approach [89], adjusting the p-values to control the false discovery rate. A simple linear model was fitted to estimate the log-ratios of each gene and an empirical Bayes method was used to assess whether genes were differentially expressed. After ranking, genes with a P-value less than 0.05 were provisionally accepted as having altered expression. P-values were adjusted for multiple testing using the procedure of Benjamini and Hochberg [91]. Eventually, only those genes whose log ratio was >1 or <-1 were taken in consideration as differentially expressed.

Differentially expressed genes were analysed for GO terms enrichment using the software GOAT [33,92].

For DDB ID's and gene description look at the dictyBase [36]. The gene identifiers correspond to the uncurated gene predictions from the Sequencing Center rather than the dictyBase curated identifiers. In most cases, and in particular for all the genes described in the text, this does not make any difference.

The DNA array experiment has been loaded into ArrayExpress with the name: Genome-wide transcriptional changes induced by phagocytosis or growth on bacteria in *Dictyostelium*. Accession: E-TABM-415. Specified release date: 2009-01-31.

## Authors' contributions

AS performed the microarray experiments, analysed the data and prepared figures and tables. GB, JS and AI designed and constructed the microarrays and contributed analysis and informatic tools. GB contributed in the designing of the array experiments and in the analysis of data and helped in drafting the paper. JS helped in performing the microarray experiments. AB carried out phagocytosis and growth experiments and prepared the RNA's. ABalbo and BP performed the Northern blot experiments and the densitometric analysis. BPeracino was responsible for gene cloning and preparation of DNA probes. SB conceived the study, helped in designing the experiments and analysing the data and drafted the manuscript.

## Supplementary Material

Additional file 1 Complete list of genes regulated by phagocytosis (cluster 1/5/7). The genes are grouped in categories, according to the categorization scheme for *D. discoideum *that is based on the yeast classification scheme [37]. The DDB number of each gene is indicated on the first column (information can be collected by connecting to the dictyBase [36]), followed by the annotation. Annotations without acronyms in italics indicate genes, whose protein products are not unambiguously identified or are unknown. Values of log2 ratio for comparisons A, B or C (see Table 1) are shown in columns 3, 4 or 5, respectively. Positive values (up-regulation) are in yellow, negative values (down-regulation) in grey. Click here for file

Additional file 2 Complete list of genes regulated by phagocytosis and growth on bacteria (cluste 4). The genes are grouped in categories, according to the categorization scheme for *D. discoideum *that is based on the yeast classification scheme [37]. The DDB number of each gene is indicated on the first column (information can be collected by connecting to the dictyBase [36]), followed by the annotation. Annotations without acronyms in italics indicate genes, whose protein products are not unambiguously identified or are unknown. Values of log2 ratio for comparisons A, B or C (see Table 1) are shown in columns 3, 4 or 5, respectively. Positive values (up-regulation) are in yellow, negative values (down-regulation) in grey. Click here for file

Additional file 3 Complete list of genes regulated by growth on bacteria (cluster 2/6). The genes are grouped in categories, according to the categorization scheme for *D. discoideum *that is based on the yeast classification scheme [37]. The DDB number of each gene is indicated on the first column (information can be collected by connecting to the dictyBase [36]), followed by the annotation. Annotations without acronyms in italics indicate genes, whose protein products are not unambiguously identified or are unknown. Values of log2 ratio for comparisons A, B or C (see Table 1) are shown in columns 3, 4 or 5, respectively. Positive values (up-regulation) are in yellow, negative values (down-regulation) in grey. Click here for file

Additional file 4 Enriched GO categories for genes in clusters 1/5/7 (genes regulated by phagocytosis). Additional file 4 lists the GO numbers and terms for biological processes, molecular functions, and cellular localizations that were found enriched in the GOAT analysis (see Figure 4) with the corresponding genes selected by GOAT. Click here for file

Additional file 5 Enriched GO categories for genes in cluster 4 and cluster 2/6. Additional file 5 lists the GO numbers and terms for biological processes, molecular functions, and cellular localizations that were found enriched in the GOAT analysis (see Figure 5) with the corresponding genes selected by GOAT. In cluster 4 and 2/6 no enriched GO terms were detected by the GOAT analysis for up-regulated genes. Click here for file

Additional file 6 A selection of genes from comparison D (V12M2 *vs*. AX2 growing on bacteria). The table shows all the differentially expressed genes encoding cytoskeletal proteins found in comparison D. In addition a few genes encoding putative membrane proteins are listed, which are strongly up-regulated and/or are characterized by interesting domains. A complete list of all genes in this comparison is available at ArrayExpress as indicated in the Methods section. Click here for file

Additional file 7 Genes in common between microarray and proteomic profile. List of the genes differentially regulated in the microarray, whose protein products are among the 179 proteins identified in the phagosomal proteome (35). The comparisons A-D are as in Table 1. Click here for file

## References

[B1] Cardelli J (2001). Phagocytosis and macropinocytosis in *Dictyostelium*: phosphoinositide-based processes, biochemically distinct. Traffic.

[B2] Botelho RJ, Scott CC, Grinstein S (2004). Phosphoinositide involvement in phagocytosis and phagosome maturation. Curr Top Microbiol Immunol.

[B3] Desjardins M, Houde M, Gagnon E (2005). Phagocytosis: the convoluted way from nutrition to adaptive immunity. Immunol Rev.

[B4] Maniak M, Rauchenberger R, Albrecht R, Murphy J, Gerisch G (1995). Coronin involved in phagocytosis: dynamics of particle-induced relocalization visualized by a green fluorescent protein Tag. Cell.

[B5] Peracino B, Borleis J, Jin T, Westphal M, Schwartz JM, Wu L, Bracco E, Gerisch G, Devreotes P, Bozzaro S (1998). G protein beta subunit-null mutants are impaired in phagocytosis and chemotaxis due to inappropriate regulation of the actin cytoskeleton. J Cell Biol.

[B6] Janssen KP, Schleicher M (2001). *Dictyostelium discoideum*: a genetic model system for the study of professional phagocytes. Profilin, phosphoinositides and the lmp gene family in *Dictyostelium*. Biochim Biophys Acta.

[B7] Maniak M (2002). Conserved features of endocytosis in *Dictyostelium*. Int Rev Cytol.

[B8] Clarke M, Maddera L (2006). Phagocyte meets prey: uptake, internalization, and killing of bacteria by *Dictyostelium* amoebae. Eur J Cell Biol.

[B9] Bozzaro S, Bucci C, Steinert M Phagocytosis and host-pathogen interactions in *Dictyostelium* with a look at macrophages. International Review of Cytology.

[B10] Solomon JM, Rupper A, Cardelli JA, Isberg RR (2000). Intracellular growth of *Legionella pneumophila* in *Dictyostelium discoideum*, a system for genetic analysis of host-pathogen interactions. Infect Immun.

[B11] Hagele S, Kohler R, Merkert H, Schleicher M, Hacker J, Steinert M (2000). *Dictyostelium discoideum*: a new host model system for intracellular pathogens of the genus *Legionella*. Cell Microbiol.

[B12] Skriwan C, Fajardo M, Hagele S, Horn M, Wagner M, Michel R, Krohne G, Schleicher M, Hacker J, Steinert M (2002). Various bacterial pathogens and symbionts infect the amoeba *Dictyostelium discoideum*. Int J Med Microbiol.

[B13] Pukatzki S, Kessin RH, Mekalanos JJ (2002). The human pathogen *Pseudomonas aeruginosa* utilizes conserved virulence pathways to infect the social amoeba *Dictyostelium discoideum*. Proc Natl Acad Sci USA.

[B14] Fajardo M, Schleicher M, Noegel A, Bozzaro S, Killinger S, Heuner K, Hacker J, Steinert M (2004). Calnexin, calreticulin and cytoskeleton-associated proteins modulate uptake and growth of *Legionella pneumophila* in *Dictyostelium discoideum*. Microbiology.

[B15] Steinert M, Heuner K (2005). *Dictyostelium* as host model for pathogenesis. Cell Microbiol.

[B16] Peracino B, Wagner C, Balest A, Balbo A, Pergolizzi B, Noegel AA, Steinert M, Bozzaro S (2006). Function and mechanism of action of *Dictyostelium* Nramp1 (Slc11a1) in bacterial infection. Traffic.

[B17] Weber SS, Ragaz C, Reus K, Nyfeler Y, Hilbi H (2006). *Legionella pneumophila* exploits PI(4)P to anchor secreted effector proteins to the replicative vacuole. PLoS Pathog.

[B18] Benghezal M, Fauvarque MO, Tournebize R, Froquet R, Marchetti A, Bergeret E, Lardy B, Klein G, Sansonetti P, Charette SJ, Cosson P (2006). Specific host genes required for the killing of *Klebsiella* bacteria by phagocytes. Cell Microbiol.

[B19] Hagedorn M, Soldati T (2007). Flotillin and RacH modulate the intracellular immunity of *Dictyostelium* to *Mycobacterium marinum* infection. Cell Microbiol.

[B20] Sussman R, Sussman M (1967). Cultivation of *Dictyostelium discoideum* in axenic medium. Biochem Biophys Res Commun.

[B21] Watts DJ, Ashworth JM (1970). Growth of myxameobae of the cellular slime mould *Dictyostelium discoideum* in axenic culture. Biochem J.

[B22] Vogel G, Thilo L, Schwarz H, Steinhart R (1980). Mechanism of phagocytosis in *Dictyostelium discoideum*: phagocytosis is mediated by different recognition sites as disclosed by mutants with altered phagocytotic properties. J Cell Biol.

[B23] Bozzaro S, Merkl R, Gerisch G (1987). Cell adhesion: its quantification, assay of the molecules involved, and selection of defective mutants in *Dictyostelium* and Polysphondylium. Methods Cell Biol.

[B24] Bozzaro S, Merkl R (1985). Monoclonal antibodies against *Dictyostelium* plasma membranes: their binding to simple sugars. Cell Differ.

[B25] Bracco E, Peracino B, Noegel AA, Bozzaro S (1997). Cloning and transcriptional regulation of the gene encoding the vacuolar/H+ ATPase B subunit of *Dictyostelium discoideum*. FEBS Lett.

[B26] Balbo A, Bozzaro S (2006). Cloning of *Dictyostelium* eIF6 (p27BBP) and mapping its nucle(ol)ar localization subdomains. Eur J Cell Biol.

[B27] Eichinger L, Rivero F (2006). Methods in Molecular Biology – Dictyostelium discoideum Protocols.

[B28] Eichinger L, Pachebat JA, Glockner G, Rajandream MA, Sucgang R, Berriman M, Song J, Olsen R, Szafranski K, Xu Q, Tunggal B, Kummerfeld S, Madera M, Konfortov BA, Rivero F, Bankier AT, Lehmann R, Hamlin N, Davies R, Gaudet P, Fey P, Pilcher K, Chen G, Saunders D, Sodergren E, Davis P, Kerhornou A, Nie X, Hall N, Anjard C (2005). The genome of the social amoeba *Dictyostelium discoideum*. Nature.

[B29] Van Driessche N, Shaw C, Katoh M, Morio T, Sucgang R, Ibarra M, Kuwayama H, Saito T, Urushihara H, Maeda M, Takeuchi I, Ochiai H, Eaton W, Tollett J, Halter J, Kuspa A, Tanaka Y, Shaulsky G (2002). A transcriptional profile of multicellular development in *Dictyostelium discoideum*. Development.

[B30] Iranfar N, Fuller D, Loomis WF (2003). Genome-wide expression analyses of gene regulation during early development of *Dictyostelium discoideum*. Eukaryot Cell.

[B31] Chubb JR, Bloomfield G, Xu Q, Kaller M, Ivens A, Skelton J, Turner BM, Nellen W, Shaulsky G, Kay RR, Bickmore WA, Singer RH (2006). Developmental timing in *Dictyostelium* is regulated by the Set1 histone methyltransferase. Dev Biol.

[B32] Farbrother P, Wagner C, Na J, Tunggal B, Morio T, Urushihara H, Tanaka Y, Schleicher M, Steinert M, Eichinger L (2006). *Dictyostelium* transcriptional host cell response upon infection with *Legionella*. Cell Microbiol.

[B33] Xu Q, Shaulsky G (2005). GOAT: An R Tool for Analysing Gene Ontologytrade mark Term Enrichment. Appl Bioinformatics.

[B34] Harris MA, Clark J, Ireland A, Lomax J, Ashburner M, Foulger R, Eilbeck K, Lewis S, Marshall B, Mungall C, Richter J, Rubin GM, Blake JA, Bult C, Dolan M, Drabkin H, Eppig JT, Hill DP, Ni L, Ringwald M, Balakrishnan R, Cherry JM, Christie KR, Costanzo MC, Dwight SS, Engel S, Fisk DG, Hirschman JE, Hong EL, Nash RS (2004). The Gene Ontology (GO) database and informatics resource. Nucleic Acids Res.

[B35] Gotthardt D, Blancheteau V, Bosserhoff A, Ruppert T, Delorenzi M, Soldati T (2006). Proteomics fingerprinting of phagosome maturation and evidence for the role of a Galpha during uptake. Mol Cell Proteomics.

[B36] dictyBase Home. http://www.dictybase.org.

[B37] Urushihara H, Morio T, Saito T, Kohara Y, Koriki E, Ochiai H, Maeda M, Williams JG, Takeuchi I, Tanaka Y (2004). Analyses of cDNAs from growth and slug stages of *Dictyostelium discoideum*. Nucleic Acids Res.

[B38] Escalante R, Sastre L (1998). A Serum Response Factor homolog is required for spore differentiation in *Dictyostelium*. Development.

[B39] Anjard C, Loomis WF (2002). Evolutionary analyses of ABC transporters of *Dictyostelium discoideum*. Eukaryot Cell.

[B40] Favard-Sereno C, Ludosky MA, Ryter A (1981). Freeze-fracture study of phagocytosis in *Dictyostelium discoideum*. J Cell Sci.

[B41] Gatfield J, Pieters J (2000). Essential role for cholesterol in entry of mycobacteria into macrophages. Science.

[B42] Peyron P, Bordier C, N'Diaye EN, Maridonneau-Parini I (2000). Nonopsonic phagocytosis of *Mycobacterium kansasii* by human neutrophils depends on cholesterol and is mediated by CR3 associated with glycosylphosphatidylinositol-anchored proteins. J Immunol.

[B43] Kannan S, Audet A, Huang H, Chen LJ, Wu M (2008). Cholesterol-rich membrane rafts and Lyn are involved in phagocytosis during *Pseudomonas aeruginosa* infection. J Immunol.

[B44] Rodriguez NE, Gaur U, Wilson ME (2006). Role of caveolae in *Leishmania chagasi* phagocytosis and intracellular survival in macrophages. Cell Microbiol.

[B45] De Chastellier C, Thilo L (2006). Cholesterol depletion in *Mycobacterium avium*-infected macrophages overcomes the block in phagosome maturation and leads to the reversible sequestration of viable mycobacteria in phagolysosome-derived autophagic vacuoles. Cell Microbiol.

[B46] Schatzle J, Bush J, Cardelli J (1992). Molecular cloning and characterization of the structural gene coding for the developmentally regulated lysosomal enzyme, alpha-mannosidase, in *Dictyostelium discoideum*. J Biol Chem.

[B47] Da Lage JL, Danchin EG, Casane D (2007). Where do animal alpha-amylases come from? An interkingdom trip. FEBS Lett.

[B48] Ashworth JM, Quance J (1972). Enzyme synthesis in myxamoebae of the cellular slime mould *Dictyostelium discoideum* during growth in axenic culture. Biochem J.

[B49] Dimond RL, Mayer M, Loomis WF (1976). Characterization and developmental regulation of beta-galactosidase isozymes in *Dictyostelium discoideum*. Dev Biol.

[B50] Mueller I, Subert N, Otto H, Herbst R, Ruhling H, Maniak M, Leippe M (2005). A *Dictyostelium* mutant with reduced lysozyme levels compensates by increased phagocytic activity. J Biol Chem.

[B51] Anjard C, Loomis WF (2006). GABA induces terminal differentiation of *Dictyostelium* through a GABAB receptor. Development.

[B52] Underhill DM, Ozinsky A (2002). Phagocytosis of microbes: complexity in action. Annu Rev Immunol.

[B53] Vega VL, De Maio A (2005). Increase in phagocytosis after geldanamycin treatment or heat shock: role of heat shock proteins. J Immunol.

[B54] Shen QH, Saijo Y, Mauch S, Biskup C, Bieri S, Keller B, Seki H, Ulker B, Somssich IE, Schulze-Lefert P (2007). Nuclear activity of MLA immune receptors links isolate-specific and basal disease-resistance responses. Science.

[B55] Wienke DC, Knetsch ML, Neuhaus EM, Reedy MC, Manstein DJ (1999). Disruption of a dynamin homologue affects endocytosis, organelle morphology, and cytokinesis in *Dictyostelium discoideum*. Mol Biol Cell.

[B56] Tse SM, Furuya W, Gold E, Schreiber AD, Sandvig K, Inman RD, Grinstein S (2003). Differential role of actin, clathrin, and dynamin in Fc gamma receptor-mediated endocytosis and phagocytosis. J Biol Chem.

[B57] Eichinger L, Lee SS, Schleicher M (1999). *Dictyostelium* as model system for studies of the actin cytoskeleton by molecular genetics. Microsc Res Tech.

[B58] Noegel AA, Schleicher M (2000). The actin cytoskeleton of *Dictyostelium*: a story told by mutants. J Cell Sci.

[B59] Soldati T (2003). Unconventional myosins, actin dynamics and endocytosis: a menage a trois?. Traffic.

[B60] Hitt AL, Iijima-Shimizu M, DuBay MJ, Antonette LL, Urushihara H, Wilkerson CG (2003). Identification of a second member of the ponticulin gene family and its differential expression pattern. Biochim Biophys Acta.

[B61] Aiba K, Fang H, Yamaguchi N, Tanaka Y, Urushihara H (1997). Isoforms of gp138, a cell-fusion related protein in *Dictyostelium discoideum*. J Biochem.

[B62] Bozzaro S, Roseman S (1983). Adhesion of *Dictyostelium discoideum* cells to carbohydrates immobilized in polyacrylamide gels. I. Evidence for three sugar-specific cell surface receptors. J Biol Chem.

[B63] Ceccarelli A, Bozzaro S (1992). Selection of mutants defective in binding to immobilized carbohydrates in *Dictyostelium discoideum*. Anim Biol.

[B64] Artavanis-Tsakonas K, Love JC, Ploegh HL, Vyas JM (2006). Recruitment of CD63 to *Cryptococcus neoformans* phagosomes requires acidification. Proc Natl Acad Sci USA.

[B65] Chang Y, Finnemann SC (2007). Tetraspanin CD81 is required for the alpha v beta5-integrin-dependent particle-binding step of RPE phagocytosis. J Cell Sci.

[B66] Burch-Smith TM, Schiff M, Caplan JL, Tsao J, Czymmek K, Dinesh-Kumar SP (2007). A novel role for the TIR domain in association with pathogen-derived elicitors. PLoS Biol.

[B67] McCoy CE, O'Neill LA (2008). The role of toll-like receptors in macrophages. Front Biosci.

[B68] Sheen J, He P (2007). Nuclear actions in innate immune signaling. Cell.

[B69] Chen G, Zhuchenko O, Kuspa A (2007). Immune-like phagocyte activity in the social amoeba. Science.

[B70] Berger P, Bonneick S, Willi S, Wymann M, Suter U (2002). Loss of phosphatase activity in myotubularin-related protein 2 is associated with Charcot-Marie-Tooth disease type 4B1. Hum Mol Genet.

[B71] Merlot S, Meili R, Pagliarini DJ, Maehama T, Dixon JE, Firtel RA (2003). A PTEN-related 5-phosphatidylinositol phosphatase localized in the Golgi. J Biol Chem.

[B72] Morgan CP, Insall R, Haynes L, Cockcroft S (2004). Identification of phospholipase B from *Dictyostelium discoideum* reveals a new lipase family present in mammals, flies and nematodes, but not yeast. Biochem J.

[B73] Seastone DJ, Zhang L, Buczynski G, Rebstein P, Weeks G, Spiegelman G, Cardelli J (1999). The small Mr Ras-like GTPase Rap1 and the phospholipase C pathway act to regulate phagocytosis in *Dictyostelium discoideum*. Mol Biol Cell.

[B74] Rupper A, Cardelli J (2001). Regulation of phagocytosis and endo-phagosomal trafficking pathways in *Dictyostelium discoideum*. Biochim Biophys Acta.

[B75] Loovers HM, Kortholt A, de Groote H, Whitty L, Nussbaum RL, van Haastert PJ (2007). Regulation of phagocytosis in *Dictyostelium* by the inositol 5-phosphatase OCRL homolog Dd5P4. Traffic.

[B76] Dormann D, Weijer G, Dowler S, Weijer CJ (2004). In vivo analysis of 3-phosphoinositide dynamics during *Dictyostelium* phagocytosis and chemotaxis. J Cell Sci.

[B77] Prabhu Y, Eichinger L (2006). The *Dictyostelium* repertoire of seven transmembrane domain receptors. Eur J Cell Biol.

[B78] Pin JP, Galvez T, Prezeau L (2003). Evolution, structure, and activation mechanism of family 3/C G-protein-coupled receptors. Pharmacol Ther.

[B79] Prabhu Y, Muller R, Anjard C, Noegel AA (2007). GrlJ, a *Dictyostelium* GABAB-like receptor with roles in post-aggregation development. BMC Dev Biol.

[B80] Little KD, Hemler ME, Stipp CS (2004). Dynamic regulation of a GPCR-tetraspanin-G protein complex on intact cells: central role of CD81 in facilitating GPR56-Galpha q/11 association. Mol Biol Cell.

[B81] Muller-Taubenberger A, Lupas AN, Li H, Ecke M, Simmeth E, Gerisch G (2001). Calreticulin and calnexin in the endoplasmic reticulum are important for phagocytosis. Embo J.

[B82] Clarke M, Kohler J, Arana Q, Liu T, Heuser J, Gerisch G (2002). Dynamics of the vacuolar H(+)-ATPase in the contractile vacuole complex and the endosomal pathway of *Dictyostelium* cells. J Cell Sci.

[B83] Rupper A, Grove B, Cardelli J (2001). Rab7 regulates phagosome maturation in *Dictyostelium*. J Cell Sci.

[B84] Chia CP, Gomathinayagam S, Schmaltz RJ, Smoyer LK (2005). Glycoprotein gp130 of *dictyostelium discoideum* influences macropinocytosis and adhesion. Mol Biol Cell.

[B85] Bozzaro S, Gerisch G (1978). Contact sites in aggregating cells of *Polysphondylium pallidum*. J Mol Biol.

[B86] Sambrook J, Maniatis T, Fritsch ET (1989). Molecular cloning: a laboratory manual.

[B87] Morio T, Urushihara H, Saito T, Ugawa Y, Mizuno H, Yoshida M, Yoshino R, Mitra BN, Pi M, Sato T, Takemoto K, Yasukawa H, Williams J, Maeda M, Takeuchi I, Ochiai H, Tanaka Y (1998). The *Dictyostelium* developmental cDNA project: generation and analysis of expressed sequence tags from the first-finger stage of development. DNA Res.

[B88] Gentleman RC, Carey VJ, Bates DM, Bolstad B, Dettling M, Dudoit S, Ellis B, Gautier L, Ge Y, Gentry J, Hornik K, Hothorn T, Huber W, Iacus S, Irizarry R, Leisch F, Li C, Maechler M, Rossini AJ, Sawitzki G, Smith C, Smyth G, Tierney L, Yang JY, Zhang J (2004). Bioconductor: open software development for computational biology and bioinformatics. Genome Biol.

[B89] Smyth GK (2004). Linear models and empirical bayes methods for assessing differential expression in microarray experiments. Stat Appl Genet Mol Biol.

[B90] Kooperberg C, Fazzio TG, Delrow JJ, Tsukiyama T (2002). Improved background correction for spotted DNA microarrays. J Comput Biol.

[B91] Benjamini Y, Hochberg Y (1995). Controlling the false discovery rate: a pratical and powerful approach to multiple testing. J R Stat Soc.

[B92] Download the GOAT package. http://dictygenome.bcm.tmc.edu/Downloads/GOAT.

